# Causal Information Approach to Partial Conditioning in Multivariate Data Sets

**DOI:** 10.1155/2012/303601

**Published:** 2012-05-21

**Authors:** D. Marinazzo, M. Pellicoro, S. Stramaglia

**Affiliations:** ^1^Department of Data Analysis, Faculty of Psychology and Pedagogical Sciences, University of Gent, 9000 Gent, Belgium; ^2^Dipartimento Interateneo di Fisica “Michelangelo Merlin”, University of Bari, 70126 Bari, Italy; ^3^TIRES-Center of Innovative Technologies for Signal Detection and Processing, University of Bari, 70125 Bari, Italy; ^4^INFN, Sezione di Bari, 70125 Bari, Italy

## Abstract

When evaluating causal influence from one time series to another in a multivariate data set it is necessary to take into account the conditioning effect of the other variables. In the presence of many variables and possibly of a reduced number of samples, full conditioning can lead to computational and numerical problems. In this paper, we address the problem of partial conditioning to a limited subset of variables, in the framework of information theory. The proposed approach is tested on simulated data sets and on an example of intracranial EEG recording from an epileptic subject. We show that, in many instances, conditioning on a small number of variables, chosen as the most informative ones for the driver node, leads to results very close to those obtained with a fully multivariate analysis and even better in the presence of a small number of samples. This is particularly relevant when the pattern of causalities is sparse.

## 1. Introduction

Determining how the brain is connected is a crucial point in neuroscience. To gain better understanding of which neurophysiological processes are linked to which brain mechanisms, structural connectivity in the brain can be complemented by the investigation of statistical dependencies between distant brain regions (functional connectivity) or of models aimed to elucidate drive-response relationships (effective connectivity). Advances in imaging techniques guarantee an immediate improvement in our knowledge of structural connectivity. A constant computational and modelling effort has to be done in order to optimize and adapt functional and effective connectivity to the qualitative and quantitative changes in data and physiological applications. The paths of information flow throughout the brain can shed light on its functionality in health and pathology. Every time that we record brain activity we can imagine that we are monitoring the activity at the nodes of a network. This activity is dynamical and sometimes chaotic. Dynamical networks [1] model physical and biological behaviour in many applications; also, synchronization in dynamical network is influenced by the topology of the network itself [2]. A great need exists for the development of effective methods of inferring network structure from time series data; a dynamic version of the Bayesian networks has been proposed in [3]. A method for detecting the topology of dynamical networks, based on chaotic synchronization, has been proposed in [4].

Granger causality has become the method of choice to determine whether and how two time series exert causal influences on each other [5, 6]. This approach is based on prediction: if the prediction error of the first time series is reduced by including measurements from the second one in the linear regression model, then the second time series is said to have a causal influence on the first one. This frame has been used in many fields of science, including neural systems [7–10], reochaos [11], and cardiovascular variability [12].

From the beginning [13, 14], it has been known that if two signals are influenced by third one that is not included in the regressions, this leads to spurious causalities, so an extension to the multivariate case is in order. The conditional Granger causality analysis (CGCA) [15] is based on a straightforward expansion of the autoregressive model to a general multivariate case including all measured variables. CGCA has been proposed to correctly estimate coupling in multivariate data sets [16–19]. Sometimes though, a fully multivariate approach can entrain problems that can be purely computational or even conceptual: in the presence of redundant variables the application of the standard analysis leads to underestimation of causalities [20].

Several approaches have been proposed in order to reduce dimensionality in multivariate sets, relying on generalized variance [16], principal components analysis [19], or the Granger causality itself [21].

In this paper we will address the problem of partial conditioning to a limited subset of variables, in the framework of information theory. Intuitively, one may expect that conditioning on a small number of variables should be sufficient to remove indirect interactions if the connectivity pattern is sparse. We will show that this subgroup of variables might be chosen as the most informative for the driver variable and describe the application to simulated examples and a real data set.

## 2. Materials and Methods

We start by describing the connection between the Granger causality and information-theoretic approaches like the transfer entropy in [22]. Let {*ξ*_*n*_}_*n*=1,…,*N*+*m*_  be a time series that may be approximated by a stationary Markov process of order *m*, that is, *p*(*ξ*_*n*_ | *ξ*_*n*−1_,…, *ξ*_*n*−*m*_) = *p*(*ξ*_*n*_ | *ξ*_*n*−1_,…, *ξ*_*n*−*m*−1_). We will use the shorthand notation *X*_*i*_ = (*ξ*_*i*_,…,*ξ*_*i*+*m*−1_)^*⊤*^ and *x*_*i*_ = *ξ*_*i*+*m*_, for *i* = 1,…, *N*, and treat these quantities as *N* realizations of the stochastic variables *X* and *x*. The minimizer of the risk functional

(1) $$\begin{array}{c} R\left[f\right]=\int dX\;dx{\left(x-f\left(X\right)\right)}^{2}p\left(X,x\right) \end{array}$$

represents the best estimate of *x*, given X, and corresponds [23] to the regression function f*(*X*) = ∫*dxp*(*x* | *X*)*x*. Now, let {*η*_*n*_}_*n*=1,…,*N*+*m*_ be another time series of simultaneously acquired quantities, and denote *Y*_*i*_ = (*η*_*i*_,…,*η*_*i*+*m*−1_)^*⊤*^. The best estimate of *x*, given *X* and *Y*, is now g*(*X*, *Y*) = ∫*dxp*(*x* | *X*, *Y*)*x*. If the generalized Markov property holds, that is,

(2) $$\begin{array}{cc} & p\left(x\;|\;X,Y\right)=p\left(x\;|\;X\right), \end{array}$$

then f*(*X*) = g*(*X*, *Y*) and the knowledge of *Y* does not improve the prediction of *x*. Transfer entropy [22] is a measure of the violation of 2: it follows that the Granger causality implies nonzero transfer entropy [24]. Under the Gaussian assumption it can be shown that the Granger causality and transfer entropy are entirely equivalent and just differ for a factor two [25]. The generalization of the Granger causality to a multivariate fashion, described in the following, allows the analysis of dynamical networks [26] and to discern between direct and indirect interactions.

Let us consider *n* time series {*x*_*α*_(*t*)}_*α*=1,…,*n*_; the state vectors are denoted:

(3) $$\begin{array}{cc} & X_{\alpha }\left(t\right)=\left(x_{\alpha }\left(t-m\right),\ldots ,x_{\alpha }\left(t-1\right)\right), \end{array}$$

*m* being the window length (the choice of *m* can be done using the standard cross-validation scheme). Let *ϵ*(*x*_*α*_ | **X**) be the mean squared error prediction of *x*_*α*_ on the basis of all the vectors **X** (corresponding to linear regression or nonlinear regression by the kernel approach described in [24]). The multivariate Granger causality index *c*(*β* → *α*) is defined as follows: consider the prediction of *x*_*α*_ on the basis of all the variables but *X*_*β*_ and the prediction of *x*_*α*_ using all the variables, then the causality measures the variation of the error in the two conditions, that is,

(4) $$\begin{array}{c} c\left(\beta \to \alpha \right)=\log\frac{\epsilon \left(x_{\alpha }\;|\;X\setminus X_{\beta }\right)}{\epsilon \left(x_{\alpha }\;|\;X\right)}. \end{array}$$

Note that in [24] a different definition of causality has been used,

(5) $$\delta \left(\beta \to \alpha \right)=\frac{\epsilon \left(x_{\alpha }\;|\;X\setminus X_{\beta }\right)-\epsilon \left(x_{\alpha }\;|\;X\right)}{\epsilon \left(x_{\alpha }\;|\;X\setminus X_{\beta }\right)}.$$

The two definitions are clearly related by a monotonic transformation:

(6) c(β→α)=−log⁡⁡[1−δ(β→α)].

Here, we first evaluate the causality *δ*(*β* → *α*) using the selection of significative eigenvalues described in [26] to address the problem of overfitting in (5); then we use (6) and express our results in terms of *c*(*β* → *α*) because it is with this definition that causality is twice the transfer entropy, equal to *I*{*x*_*α*_; *X*_*β*_ | **X**∖*X*_*β*_}, in the Gaussian case [25].

Turning now to the central point of this paper, we address the problem of coping with a large number of variables, when the application of the multivariate Granger causality may be questionable or even unfeasible, whilst bivariate causality would detect also indirect causalities. Here, we show that conditioning on a small number of variables, chosen as the most informative for the candidate driver variable, is sufficient to remove indirect interactions for sparse connectivity patterns. Conditioning on a large number of variables requires a high number of samples in order to get reliable results. Reducing the number of variables, that one has to condition over, would thus provide better results for small data sets. In the general formulation of the Granger causality, one has no way to choose this reduced set of variables; on the other hand, in the framework of information theory, it is possible to individuate the most informative variables one by one. Once that it has been demonstrated [25] that the Granger causality is equivalent to the information flow between the Gaussian variables, partial conditioning becomes possible for the Granger causality estimation; to our knowledge this is the first time that such approach is proposed.

Concretely, let us consider the causality *β* → *α*; we fix the number of variables, to be used for conditioning, equal to *n*_*d*_. We denote by **Z** = (*X*_*i*_1__,…, *X*_*i*_*n*_*d*___) the set of the *n*_*d*_ variables, in **X**∖*X*_*β*_, most informative for *X*_*β*_. In other words, **Z** maximizes the mutual information *I*{*X*_*β*_; **Z**} among all the subsets **Z** of *n*_*d*_ variables. Then, we evaluate the causality

(7) $$\begin{array}{cc} & c\left(\beta \to \alpha \right)=\log\frac{\epsilon \left(x_{\alpha }\;|\;Z\right)}{\epsilon \left(x_{\alpha }\;|\;Z\cup X_{\beta }\right)}. \end{array}$$

Under the Gaussian assumption, the mutual information *I*{*X*_*β*_; **Z**} can be easily evaluated, see [25]. Moreover, instead of searching among all the subsets of *n*_*d*_ variables, we adopt the following approximate strategy. Firstly, the mutual information of the driver variable, and each of the other variables, is estimated, in order to choose the first variable of the subset. The second variable of the subsets is selected among the remaining ones as those that, jointly with the previously chosen variable, maximize the mutual information with the driver variable. Then, one keeps adding the rest of the variables by iterating this procedure. Calling **Z**_*k*−1_ the selected set of *k* − 1 variables, the set **Z**_*k*_ is obtained adding, to **Z**_*k*−1_, the variable, among the remaining ones, with the greatest information gain. This is repeated until *n*_*d*_ variables are selected. This greedy algorithm, for the selection of relevant variables, is expected to give good results under the assumption of sparseness of the connectivity.

## 3. Results and Discussion

### 3.1. Simulated Data

**Figure 1:**
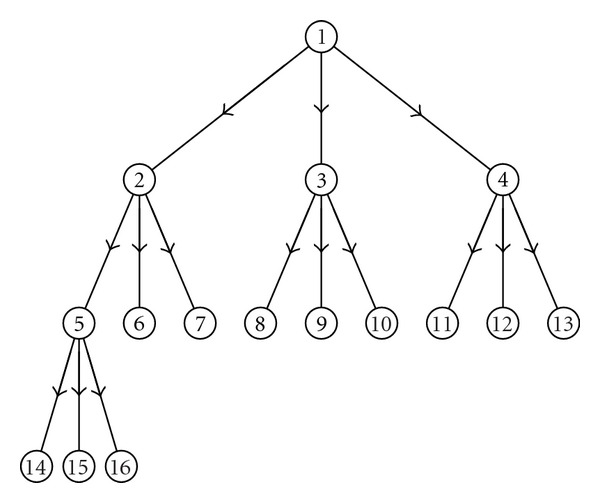
A directed rooted tree of 16 nodes.

**Figure 2:**
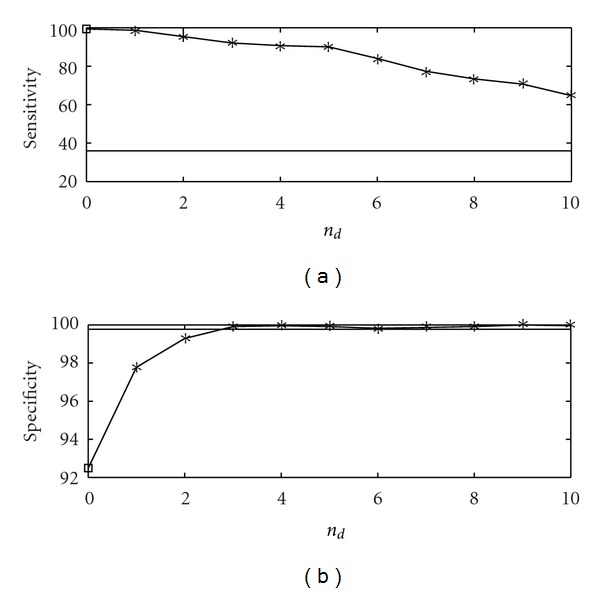
The sensitivity (a) and the specificity (b) are plotted versus *n*_*d*_, the number of variables selected for conditioning, for the first example, the rooted tree. The number of samples *N* is 100, and the order is *m* = 2; similar results are obtained varying *m*. The results are averaged over 100 realizations of the linear dynamical system described in the text. The empty square, in correspondence to *n*_*d*_ = 0, is the result from the bivariate analysis. The horizontal line is the outcome from multivariate analysis, where all variables are used for conditioning.

 Let us consider linear dynamical systems on a lattice of *n* nodes, with equations, for *i* = 1,…, *n*

(8) $$\begin{array}{c} x_{i,t}=\overset{n}{\underset{j=1}{\sum }}a_{ij}x_{j,t-1}+s\tau_{i,t}, \end{array}$$

where *a*'s are the couplings, *s* is the strength of the noise, and *τ*'s are unit variance i.i.d. Gaussian noise terms. The level of noise determines the minimal amount of samples needed to assess that the structures recovered by the proposed approach are genuine and are not due to randomness as it happens for the standard Granger causality (see discussions in [24, 26]); in particular noise should not be too high to obscure deterministic effects. Firstly we consider a directed tree of 16 nodes depicted in Figure 1; we set *a*_*ij*_ equal to 0.9 for each directed link of the graph thus obtained and zero otherwise. We set *s* = 0.1. In Figure 2 we show the application of the proposed methodology to data sets generated by (8), 100 samples long, in terms of quality of the retrieved network, expressed in terms of sensitivity (the percentage of existing links that are detected) and specificity (the percentage of missing links that are correctly recognized as nonexisting). The bivariate analysis provides 100% sensitivity and 92% specificity. However, conditioning on a few variables is sufficient to put in evidence just the direct causalities while still obtaining high values of sensitivity. The full multivariate analysis (obtained as *n*_*d*_ tends to 16) gives here a rather low sensitivity due to the low number of samples. This is a clear example where conditioning on a small number of variables is better than conditioning on all the variables at hand.

**Figure 3:**
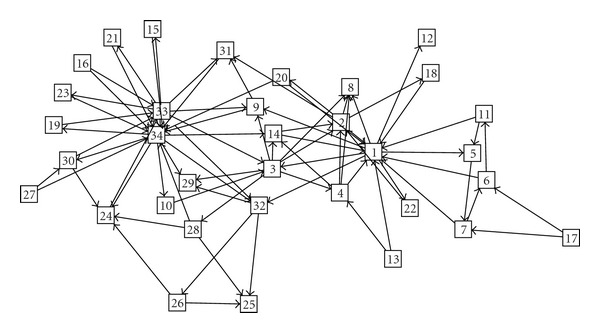
The directed network of 34 nodes obtained assigning randomly a direction to links of the Zachary network.

As another example, we now fix *n* = 34 and construct couplings in terms of the well-known Zachary data set [27], an undirected network of 34 nodes. We assign a direction to each link, with equal probability, and set *a*_*ij*_ equal to 0.015, for each link of the directed graph thus obtained, and zero otherwise. The noise level is set as *s* = 0.5. The network is displayed in Figure 3: the goal is again to estimate this directed network from the measurements of time series on nodes.

**Figure 4:**
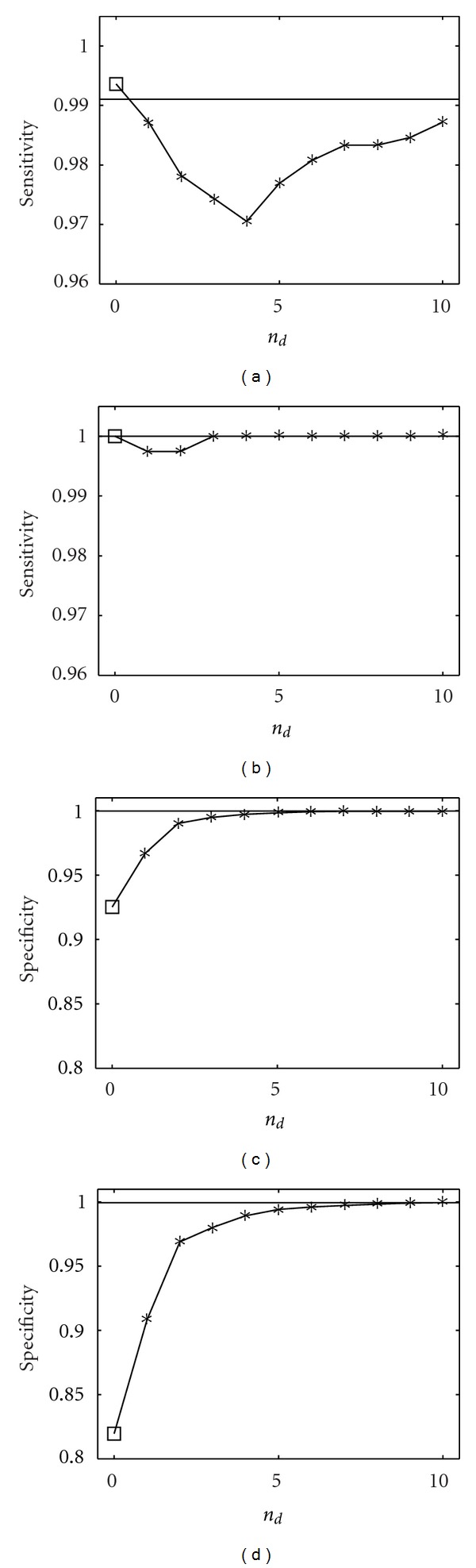
Sensitivity and specificity are plotted versus *n*_*d*_, the number of variables selected for conditioning, for two values of the number of samples *N*, 500 (left) and 1000 (right). The order is *m* = 2, similar results are obtained varying *m*. The results are averaged over 100 realizations of the linear dynamical system described in the text. The empty square, in correspondence to *n*_*d*_ = 0, is the result from the bivariate analysis. The horizontal line is the outcome from multivariate analysis, where all variables are used for conditioning.

**Figure 5:**
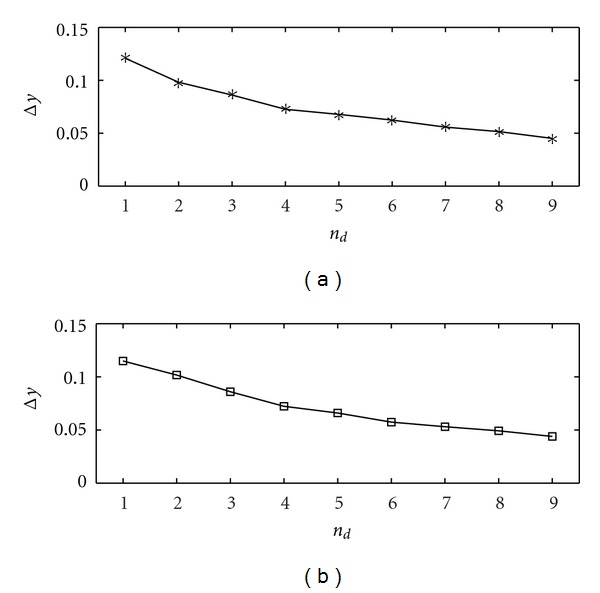
The mutual information gain, when the (*n*_*d*_ + 1)th variable is included, is plotted versus *n*_*d*_ for two values of the number of samples *N*, 500 (a) and 1000 (b). The order is *m* = 2. The information gain is averaged over all the variables.

In Figure 4 we show the application of the proposed methodology to data sets generated by (8), in terms of sensitivity and specificity, for different numbers of samples. The bivariate analysis detects several false interactions; however, conditioning on a few variables is sufficient to put in evidence just the direct causalities. Due to the sparseness of the underlying graph, we get a result that is very close to the one by the full multivariate analysis; the multivariate analysis here recovers the true network, and indeed the number of samples is sufficiently high. In Figure 5, concerning the stage of selection of variables upon which conditioning is performed, we plot the mutual information gain as a function of the number of variables included *n*_*d*_: it decreases as *n*_*d*_ increases.

### 3.2. EEG Epilepsy Data

**Figure 6:**
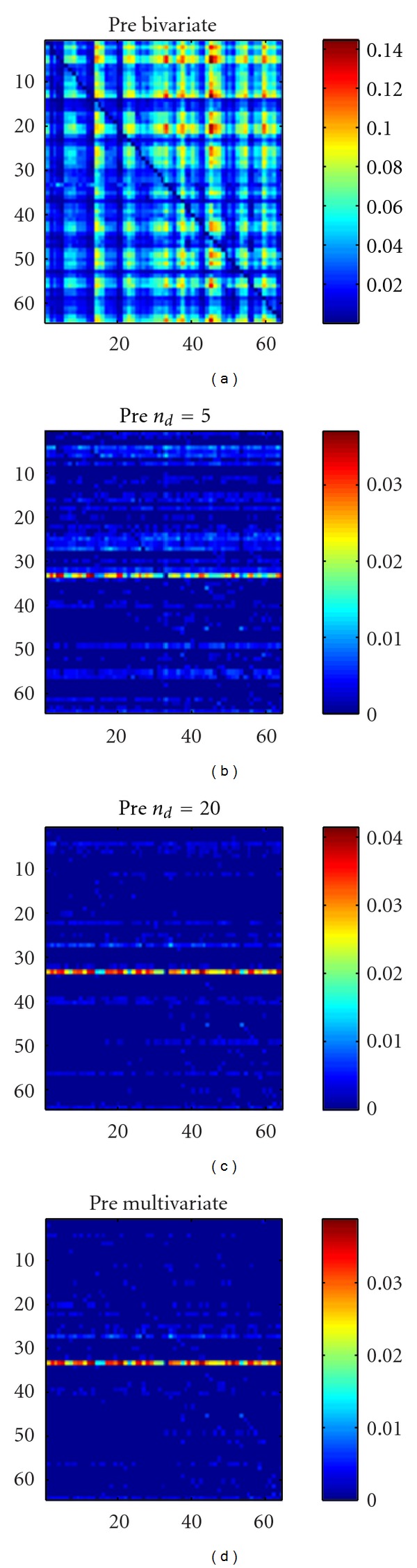
The causality analysis of the preictal period. The causality *c*(*i* → *j*) corresponds to the row *i* and the column *j*. The order is chosen as *m* = 6 according to the AIC criterion. (a) Bivariate analysis. (b) Our approach with *n*_*d*_ = 5 conditioning variables. (c) Our approach with *n*_*d*_ = 20 conditioning variables. (d) The multivariate analysis.

**Figure 7:**
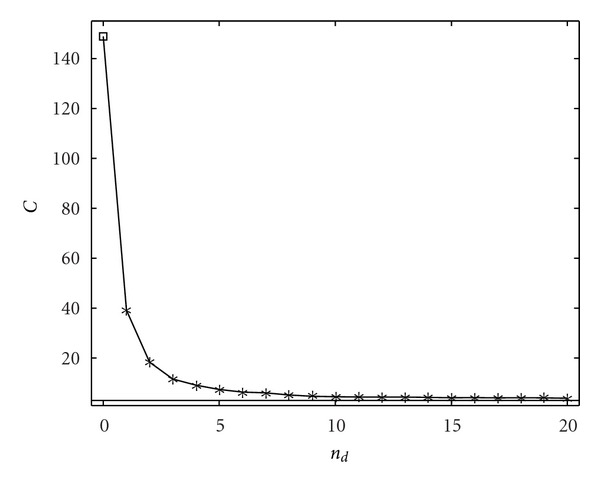
Concerning the preictal period, the sum of all causalities is plotted versus the number of conditioning variables.

 We consider now a real data set from an 8 × 8-electrode grid that was implanted in the cortical surface of the brain of a patient with epilepsy [28]. We consider two 10-second intervals prior to and immediately after the onset of a seizure, called, respectively, the preictal period and the ictal period. In Figure 6 we show the application of our approach to the preictal period; we used the linear causality. The bivariate approach detects many causalities between the electrodes; most of them, however, are indirect. According to the multivariate analysis there is just one electrode that is observed to influence the others, even in the multivariate analysis: this electrode corresponds to a localized source of information and could indicate a putative epileptic focus. In Figure 6 it is shown that conditioning on *n*_*d*_ = 5 or *n*_*d*_ = 20 variables provides the same pattern corresponding to the multivariate analysis, which thus appears to be robust. These results suggest that the effective connectivity is sparse in the preictal period. As a further confirmation, in Figure 7 we plot the sum of all causalities detected as a function of the number of conditioning variables, for the preictal period; a plateau is reached already for small values of *n*_*d*_.

**Figure 8:**
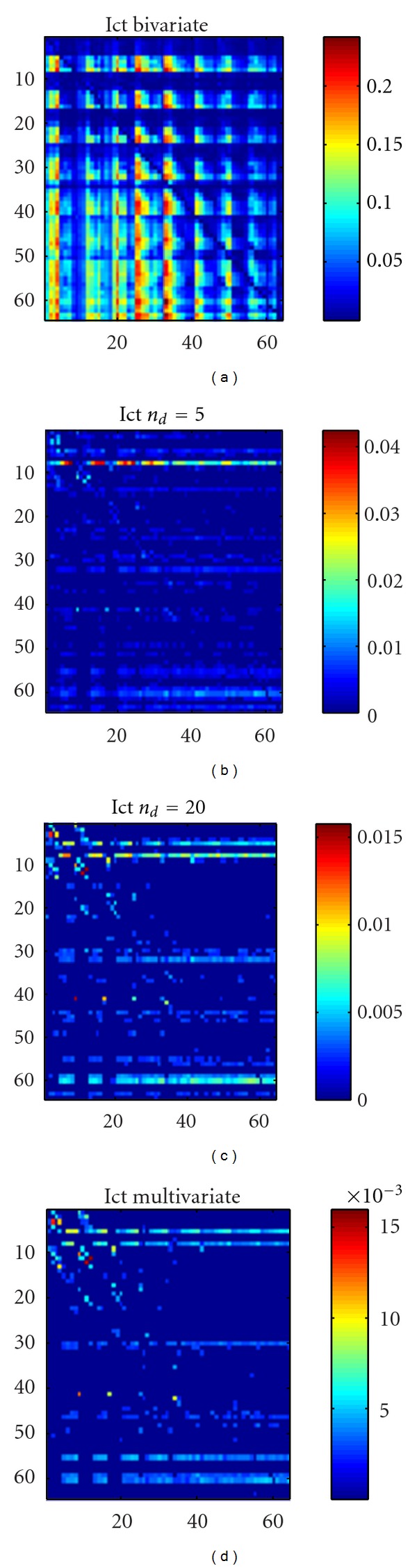
The sum of outgoing causality from each electrode in the EEG application, ictal period. (a) Bivariate analysis. (b) Our approach with *n*_*d*_ = 5 conditioning variables. (c) Our approach with *n*_*d*_ = 20 conditioning variables. (d) The multivariate analysis.

**Figure 9:**
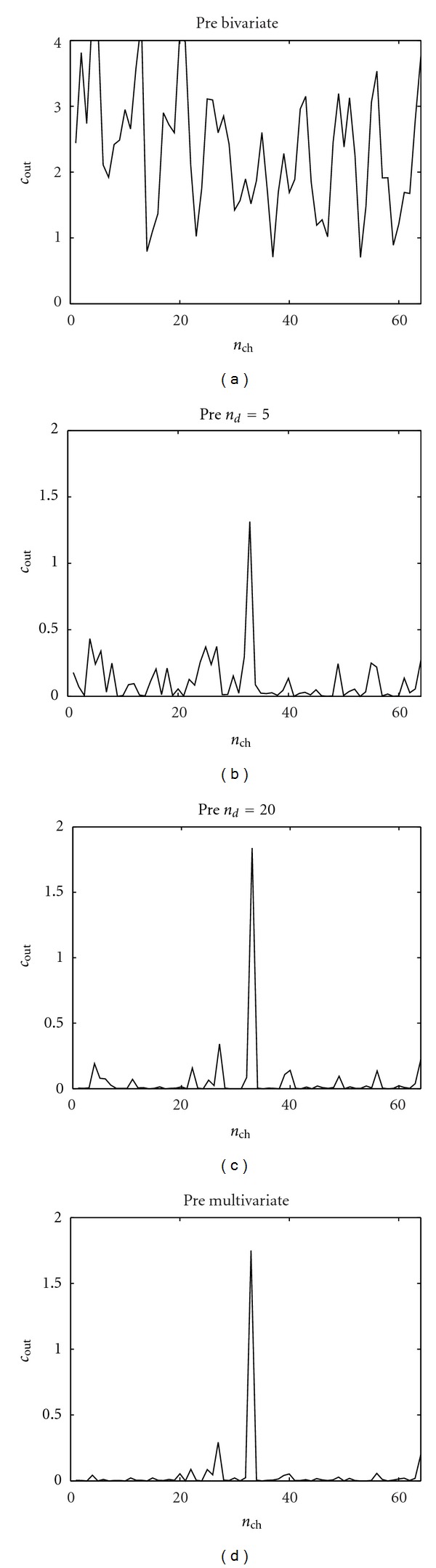
The sum of outgoing causality from each electrode in the EEG application, preictal period. (a) Bivariate analysis. (b) Our approach with *n*_*d*_ = 5 conditioning variables. (c) Our approach with *n*_*d*_ = 20 conditioning variables. (d) The multivariate analysis.

**Figure 10:**
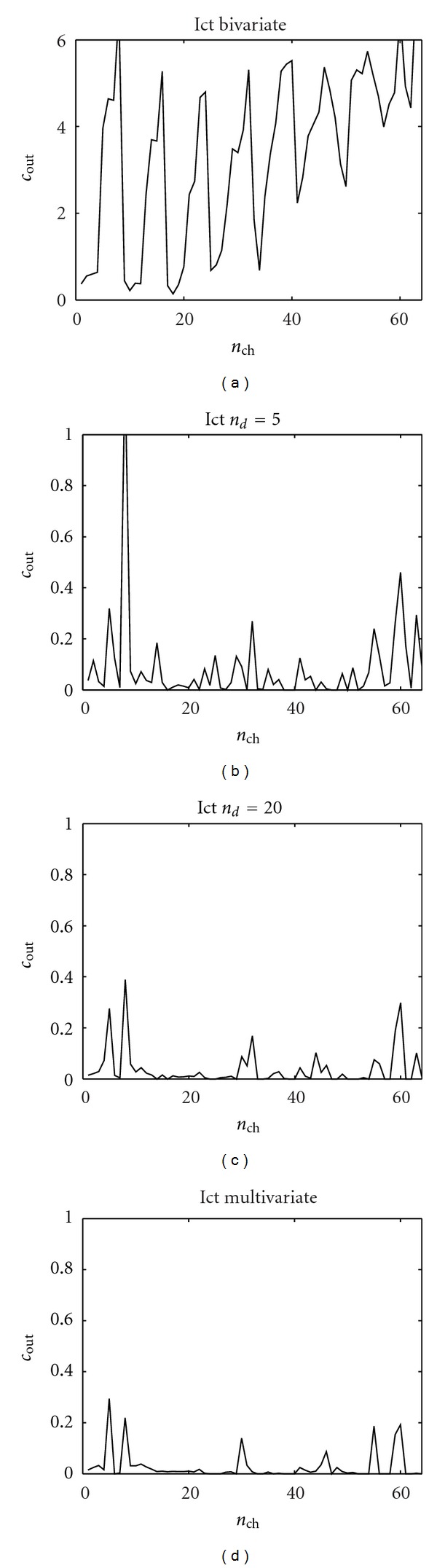
The causality analysis of the ictal period. The causality *c*(*i* → *j*) corresponds to the row *i* and the column *j*. The order is chosen as *m* = 6 according to the AIC criterion. (a) Bivariate analysis. (b) Our approach with *n*_*d*_ = 5 conditioning variables. (c) Our approach with *n*_*d*_ = 20 conditioning variables. (d) The multivariate analysis.

In Figure 8 the same analysis is shown w.r.t. the ictal period: in this case conditioning on *n*_*d*_ = 5 or *n*_*d*_ = 20 variables does not reproduce the pattern obtained with the multivariate approach. The lack of robustness of the causality pattern w.r.t. *n*_*d*_ seems to suggest that the effective connectivity pattern, during the crisis, is not sparse. In Figures 9 and 10 we show, for each electrode and for the preictal and ictal periods, respectively, the total outgoing causality (obtained as the sum of the causalities on all the other variables). These pictures confirm the discussion above: looking at how the causality changes with *n*_*d*_ may provide information about the sparseness of the effective connectivity.

## 4. Conclusions

 We have addressed the problem of partial conditioning to a limited subset of variables while estimating causal connectivity, as an alternative to full conditioning, which can lead to computational and numerical problems. Analyzing simulated examples and a real data set, we have shown that conditioning on a small number of variables, chosen as the most informative ones for the driver node, leads to results very close to those obtained with a fully multivariate analysis and even better in the presence of a small number of samples, especially when the pattern of causalities is sparse. Moreover, looking at how causality changes with the number of conditioning variables provides information about the sparseness of the connectivity.
